# Genetic Identification of Human Skeletal Remains in Forensic Context: A Review

**DOI:** 10.3390/genes17040492

**Published:** 2026-04-21

**Authors:** Laura Cainé, Madalena Henriques, Adelina Rohovska, Bárbara Sousa, Heloísa Afonso Costa, Helena Correia Dias, Joana Rodrigues, Magda Franco, Olena Mukan, Rui Nascimento, Vânia Mofreita, António Amorim

**Affiliations:** 1 National Institute of Legal Medicine and Forensic Sciences, 3000-548 Coimbra, Portugal; 2 Faculty of Medicine, University of Porto, 4200-319 Porto, Portugal; 3 LAQV REQUIMTE–Associated Laboratory for Green Chemistry and Technology, University of Porto, 4200-465 Porto, Portugal; 4 School of Sciences and Technology, University of Évora, 7004-516 Évora, Portugal; 5 NOVA School of Science and Technology, 2829-516 Caparica, Portugal; 6 Center for Research in Anthropology and Health (CIAS), Department of Life Sciences (DCV), University of Coimbra, 3000-456 Coimbra, Portugal; 7 Center for Functional Ecology (CEF), Anthropology Laboratory (LAF), Department of Life Sciences (DCV), University of Coimbra, 3000-456 Coimbra, Portugal; 8 School of Life Sciences and Environment, University of Trás-os-Montes e Alto Douro, 5000-801 Vila Real, Portugal; 9 Faculty of Sciences, University of Lisbon, 1749-016 Lisbon, Portugal; 10 Faculty of Medicine, University of Lisbon, 1649-028 Lisbon, Portugal

**Keywords:** forensics, identification, human remains, bones, teeth, mtDNA, STR, SNP

## Abstract

**Background/Objectives:** Genetic identification of human skeletal remains plays a pivotal role in forensic investigations when other traditional or primary methods are not appropriate. Decomposition, storage and environmental conditions often leave the skeletal structure as the only basis for identification. This review synthesizes current methodologies and technological advances in damaged DNA extraction and analysis, emphasizing the forensic relevance of skeletal remains for genetic identification. **Methods:** A comprehensive literature analysis highlights the basis of genetic identification; sampling that considers intrinsic and extrinsic factors influencing the DNA yield and its quality; pre-treatment methods; extraction protocols that are suitable for its sensitivity; genetic marker panels that allow for human identification; and statistical evaluation and analysis of the results. The last chapter demonstrates the real-world impact of genetic identification on historical cases, underscoring its broader significance in legal, humanitarian, and socio-historical contexts, supporting a critical evaluation of best practices, methodological robustness, and ethical considerations within the field. **Results:** Teeth, femur and the petrous portion of temporal bone are the main samples used for genetic analysis. STR profiling and mitochondrial DNA are the gold standard markers for skeletal human identification. Minimally destructive protocols that enhance a high DNA yield are chosen, with silica-based methods being highlighted in the extraction protocols. Next-Generation Sequencing techniques have also improved analytical outcomes, by enabling high-throughput data generation, increased coverage depth, nucleotide-level sequence data, and high-level multiplexing of genetic targets. **Conclusions:** This review provides a comprehensive framework for researchers and practitioners seeking to optimize genetic identification workflows in forensic sciences and bioarcheology. These methodological advances have significantly increased identification success rates, especially in cases involving degraded or limited skeletal remains. Reviews such as this one help us to identify methodological gaps, ethical concerns, and future research directions, thereby establishing best practices when working with highly degraded skeletal material, supporting more reliable, standardized, and legally defensible applications of genetic identification in forensic, archeological, and humanitarian contexts.

## 1. Introduction

Forensic investigations often deal with catastrophes that lead to missing people and unidentified skeletal remains. Whether the context is mass disaster, natural disasters, armed conflicts, refugees’ movements, terrorism acts, sexual assaults and many others, identification is crucial for forensic experts, since it is required in criminal, civil and legal issues [1,2].

There are several limitations concerning individual identification. First, gathering case information and defining the objectives, then, analyzing the remains and samples, and lastly, reconciliation between all data considered. Identification is a comparative process and depends on the condition of the remains and information available. Forensic experts separate the primary methods of identification (DNA analysis, dactyloscopy, odontology and unique medical product identification) and secondary methods (forensic anthropology, tattoos, personal accessories/belongings, personal documents—identity card, passports, etc., and antemortem records—imageology and radiology) [3,4,5,6,7]. When other primary identification methods are not applicable, genetic identification is valuable, since it provides a positive identification; presents high discrimination power (“genetic fingerprint”); can be applied to different types of samples, including degraded skeletal remains; facilitates kinship analysis and has legal and forensic acceptance. In many jurisdictions, DNA evidence is seen as highly reliable and admissible in court. Considering the success of DNA profiling, genetic identification has been widely applied [8,9].

Technological advancements have improved the genetic identification process, but severe environmental conditions and poor preservation still limit methodological accuracy. Developments related to damaged DNA extraction, PCR inhibitor removal and sequencing have been the major targets for reducing time and resource consumption. However, further improvements are needed [6,10,11,12,13,14,15].

This review focuses on the current status of genetic human identification through skeletal remains in forensic contexts, providing a comprehensive framework for researchers and practitioners seeking to optimize genetic identification workflows in forensic sciences and bioarcheology.

## 2. Methodology

### 2.1. Review Framework

To address the current status of genetic identification of human skeletal remains in a forensic context, specific topics were chosen to mention: individual genetic identification in forensic contexts; samples used for comparison; types of bone samples for genetic identification; pre-treatment methods for bone samples; methods for extracting nucleic acids from bone samples; genetic marker panels used in human identification; analysis of the results and statistical evaluation; and cases of historical and scientific relevance and ethical considerations.

### 2.2. Search Strategy

#### 2.2.1. Databases

Based on the same inclusion and exclusion criteria, each author of each chapter developed a comprehensive literature analysis for each topic, based on the following sources: the PubMed, SciELO and Web of Science databases, and books published by author John M. Butler.

#### 2.2.2. Eligibility Criteria

Inclusion criteria: The analyzed literature was narrowed through a selection process that prioritized relevance to the topic, using the keywords “genetic” AND “identification” AND “human” AND “bone” AND “remains” AND “forensic,” and key terms “ancient DNA” AND “forensic” AND “human identification” AND “remains”.

Exclusion criteria: Articles that, based on the title alone, can be deduced as not falling within the scope of our work theme/subject.

### 2.3. Data Extraction Process

Between 20 March 2025 and 2 April 2026, the following results were recovered.

For the keywords “genetic” AND “identification” AND “human” AND “bone” AND “remains” AND “forensic”, there were 237 results from PubMed, 3 results from SciELO, and 148 results from Web of Science.

For the key terms “ancient DNA” AND “forensic” AND “human identification” AND “remains”, there were 158 results from PubMed, 2 results from SciELO, and 165 results from Web of Science.

There were a total of 187 references used for this review.

**Figure 1:**
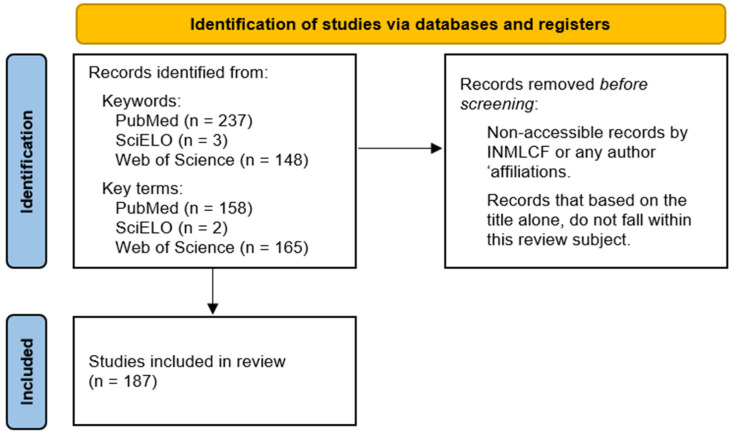
Review flowchart (based on PRISMA flow diagram [16,17]).

The following flowchart schematically describes this review framework. Considering that this study is not a systematic review, no formal systematic review protocol (e.g., PRISMA guidelines) was applied, and the original PRISMA flow diagram was not used; instead, an adapted version was created (Figure 1).

## 3. Individual Genetic Identification in Forensic Contexts

Forensic geneticists essentially deal with three main areas in their routine casework: individual identification, kinship investigation cases (essentially, paternity cases—not forgetting that there are also other kinship investigations, and even maternity), and criminal investigations.

Every year, numerous individuals are reported missing under suspicious circumstances. While some are eventually located alive through law enforcement efforts, many are discovered as unidentified human remains, which is often the result of violent crimes such as murder or sexual assault. Identifying these remains is essential for solving the cases and providing closure to the families involved [1,18,19]. The identification of an individual is one of the most relevant issues for forensic experts, and, particularly, for forensic geneticists. Identification is required in criminal, civil, and legal issues. For instance, identification is crucial for the establishment of legal responsibility, cases of refugees, mass disasters, missing persons, or criminal investigations related to sexual assaults, among others [1,2].

Positive identification is always a complex, scientific, and comparative process. It relies on methods that are capable of establishing an individual’s identity by itself; this means confirming the identity of a person (for instance, victim, aggressor, or missing person). As a comparative process, reference data are required for comparison and allow for positive identification. For individual positive identification, forensic geneticists compare the genetic profile obtained from reference samples with the genetic profile obtained from any source of DNA available, depending on the case. In forensic contexts, positive identification is achieved through primary methods of identification that allow for establishing the identity of the person without doubt [1,2]. According to Interpol, these are DNA analysis, dactyloscopy, and odontology. The surgical and medical serial number of prosthetics and other medical devices may also lead to a positive identification of an individual. Despite this, we should also consider secondary methods of identification, such as forensic anthropology, tattoos, and personal accessories (such as pieces of jewelry or wrist watches), among others. These secondary methods do not allow for positive identification by itself, but they can guide the experts to the identification process and help in the criminal investigation (e.g., they can be used as a source of DNA for comparison) [5].

DNA analysis represents one of the most accurate methods of identification in forensics and one of the most powerful sources of information for forensic cases [8,9,20]. Additionally, DNA analysis can also be used to assess several phenotypic characteristics: sex, ancestry, and age (through an epigenetic approach) [21,22]. In recent years, age estimation through DNA methylation analysis has emerged as one of the fastest-developing forensic epigenetic approaches [23]. This concept is based on the correlation between DNA methylation levels on CpG sites and chronological age [24]. Several age prediction models, named APMs, have been developed using different methodologies, such as sanger sequencing, droplet digital PCR (ddPCR), massively parallel sequencing (MPS), pyrosequencing, and SNAPshot, among others [25]. These APMs have also been developed by considering several sample types, such as blood samples, bones, teeth, saliva, buccal swabs, sperm, hair, bloodstains, among others. Furthermore, some multi-tissue APMs have also been developed, being applied to several sample types with the same accuracy in age prediction [25]. This field of research needs to be continuously investigated to improve age estimation in forensic contexts: more importantly than understanding degenerative methylation drift, there is a need to distinguish adaptive repair processes (resilience). Epigenetic age estimation has, thus, entered the field of mechanistic indicators of biological resilience, instead of remaining as a statistical predictor [24].

Forensic DNA laboratories utilize techniques based on the same fundamental principles used in medical diagnostics and genetic mapping to identify individuals. These methods allow for the extraction of an individual’s genetic profile from even minute amounts of DNA found in various biological samples such as blood, saliva, bone, hair, semen, or other tissues. All cells in the human body are derived from a single fertilized egg and contain the same DNA, with only occasional mutations causing variation. As a result, DNA extracted from any nucleated cell within the body will generally provide consistent genetic information, ensuring that forensic analysis can identify individuals based on their unique genetic makeup [26,27,28].

In investigations of missing people, genetic samples are typically divided into three types: direct reference samples, family reference samples, and samples from unidentified human remains. Direct reference samples might include previously collected biological material from the missing person, such as blood spots from newborn screening or biopsy samples. Family reference samples are generally taken from close biological relatives. Distant relatives can also provide valuable genetic material, particularly when mitochondrial or Y-chromosome DNA analysis is used, establishing maternal or paternal lineage, respectively. The combination of samples from several close relatives can improve the accuracy and reliability of kinship assessments [26,27,29,30].

Unidentified human remains samples usually consist of bones, teeth, hair or other tissues [19,26,31,32]. In cases in which remains are old or heavily degraded, these tissues often become the only biological sources available for DNA sampling [26]. However, genetic analysis can be challenging when DNA is highly degraded or present in low quantities. Despite these limitations, forensic genetics has substantially evolved over the years. In many of these samples, only trace amounts of DNA are available; therefore, the presence of hundreds or thousands of copies of mtDNA in each cell enhances the likelihood of successfully obtaining a DNA profile from mtDNA, rather than with nuclear DNA markers [26,27,33].

The gold standard for the identification and discrimination of individuals is Short Tandem Repeats (STR) profiling, which consists of the number of repeated sequences of specific DNA bases (typically, two to six base pairs) in specific DNA loci—the number of repeats of each sequence vary between individuals [34,35,36,37]. However, these methods are not always effective, even with the improvements offered by miniSTR techniques [38]. Other DNA markers such as Y-STR, mtDNA, and X-STRs should also be analyzed, helping to solve complex forensic cases, criminal assaults or challenging cases of kinship relationships [8,9,27,33,39,40].

Human identity testing through DNA analysis using STR markers has numerous applications. These include (1) determining paternity to identify a child’s biological father, (2) assisting in disaster victim identification following large-scale natural or man-made catastrophes, (3) exploring one’s heritage through genetic genealogy and ancestry testing, and (4) aiding in the investigation of historical cases and missing persons by linking unidentified human remains to their relatives [34,41]. Due to exposure to environmental conditions, these samples are most likely degraded, which makes it difficult to extract authentic DNA and obtain a complete STR profile [42]. Since we are working with low copy number (LCN) DNA, it is crucial to ensure that an accurate interpretation of the STR profile is obtained and that the peaks are from an authentic DNA sequence and not artifacts. Some authors have reported a list of artifact peaks belonging to microbes found in STR profiles from old specimen [20,43,44].

To aid the identification, in addition to STRs, there are also Next Generation Sequence (NGS) panels available to identify millions of Single Nucleotide Polymorphisms (SNPs) [45,46]. SNP markers are normally designed in small-sized amplicons, which make it easier to retrieve DNA sequences from degraded samples [20,44]. In both forensic and identification purposes, it is crucial to have a genetic profile to compare our sample to. It can either be from a suspect, the victim that needs to be identified, or a relative [47]. In cases where there are no relatives, there are some markers that can predict some externally visible (phenotypic) characteristics, such as eye, skin and hair color, and that can also give us information about the ancestry of the individual [21,48,49,50]. SNPs can be a useful tool in these cases, as they have a low mutation rate and can be compared with distant relatives [51].

Thus, the reliability of genetic identification lies in the intersection of biological uniqueness and methodological robustness. Advances in DNA extraction, amplification, and profiling technologies have further strengthened the applicability of this field across the forensic, medical, and research domains. The emerging development of quick, low-cost and accurate methods and laboratory approaches has improved the efficiency, sensitivity and discrimination power, as well as reducing the time and resource consumption of DNA analysis, providing timely answers to victims’ families [20,52,53,54].

## 4. Samples Used for Comparison

Successful genetic identification of skeletal remains requires reference samples against which to compare the DNA profile obtained from the human remains. These samples generally include personal belongings (reference samples), biological specimens from presumed relatives, and DNA profiles in forensic databases [55,56,57].

**Personal Belongings**

Personal items, such as toothbrushes, razors, hairbrushes, clothing, or frequently handled objects (e.g., eyeglasses), often retain epithelial cells, saliva, blood, and hair follicles, among others, which are suitable for DNA extraction [41,58]. When these items have a well-documented chain of custody and were used exclusively by the missing individual, they provide the most direct route for matching nuclear or mtDNA.

**Biological Relatives**

When direct reference material is unavailable, samples from close relatives can be used to make a positive identification. First-degree relatives (parents, children or full siblings) yield the highest LRs (Likelihood Ratios) when comparing autosomal STRs [59,60,61,62]. Maternal lineage can be confirmed via mtDNA comparisons, while paternal lineage relies on Y-STRs or Y-SNPs [63,64,65,66,67,68,69]. If only distant relatives are available (e.g., grandparents, maternal or paternal uncles/aunts, or cousins), additional STR or SNP panels may be necessary to achieve reliable kinship inference [51,62,70].

**Forensic DNA Databases**

National and international DNA databases store STR profiles from convicted offenders or from family reference samples in missing persons cases [71,72]. When the bone’s STR profile is uploaded, a direct match across all core loci yields a presumptive identity; a partial match may trigger a familial search, suggesting that a close relative of the database entrant is the individual in question. The utility of DNA databases in human identification has increased substantially in recent years [72], demonstrating their value not only in solving current forensic cases but also cold cases and identifying disaster victims.

The scale and global expansion of forensic DNA databases have significantly enhanced their utility in human identification. The Combined DNA Index System (CODIS) in the United States contains over 19 million offender profiles, more than 6 million arrestee profiles, and approximately 1.4 million forensic profiles at the national level (NDIS), representing one of the largest operational forensic DNA databases worldwide [73]. In Europe, national databases such as the United Kingdom National DNA Database (NDNAD) include over 7 million DNA profiles [74]. In France, the Fichier national automatisé des empreintes génétiques (FNAEG) contained approximately 7.4 million DNA profiles as of January 2025 [75]. China operates one of the largest forensic DNA databases globally. According to the published data, the Chinese national DNA database contained approximately 68 million DNA profiles as early as 2018, and has continued to expand rapidly in recent years, reflecting extensive national implementation. However, the scale and rapid expansion of this database have also raised ethical and human rights concerns, particularly regarding consent, privacy, and the collection of genetic data from specific population groups [76].

This global growth underscores the increasing role of forensic DNA databases in facilitating direct matches, familial searches, and international cooperation, particularly in missing persons cases and transnational investigations. However, to fully take advantage of the potential of these databases, it is critical to ensure their proper implementation and governance [10,57]. This includes the use of standardized protocols, rigorous quality control, and legal–ethical frameworks that protect individual rights. Additionally, the enhancement of international cooperation and data-sharing agreements between countries can significantly improve the effectiveness of transnational searches, especially in cases involving missing migrants or unidentified remains with an unknown nationality. These improvements are essential for maximizing the impact and reliability of DNA databases in forensic identification workflows [57,77].

**Forensic Genetic Genealogy**

In addition to traditional forensic DNA databases, recent advances have introduced forensic genetic genealogy (FGG) as a complementary approach for human identification. Unlike conventional STR-based databases, FGG relies on the analysis of genome-wide SNP profiles and their comparison with the profiles that are available in genealogical databases. This approach enables the identification of more distant genetic relatives, which are often beyond the resolution of traditional STR markers, and supports the reconstruction of extended family trees that can ultimately lead to the identification of unknown individuals in the absence of direct reference samples [78,79,80].

FGG has been increasingly applied in forensic investigations over the past decade, contributing to the resolution of hundreds of cases worldwide, including both criminal investigations and the identification of unidentified human remains [81,82]. This approach is particularly valuable in cases where close relatives are not available for comparison, as genome-wide SNP data enables the detection of distant familial relationships that would not be identified by using traditional markers. Notably, FGG has played a key role in resolving long-term unidentified remains cases, including several “John/Jane Doe” cases where conventional STR-based approaches failed to provide a match. One of the most well-known applications of FGG is the identification of the Golden State Killer in 2018, where investigators used genealogical databases to identify distant relatives and reconstruct family trees leading to the suspect [83]. Since this landmark case, similar methodologies have been successfully applied to cold cases and unidentified skeletal remains, demonstrating the growing relevance of FGG in forensic identification [84,85,86,87,88].

However, its effectiveness depends on database coverage and population representation, and it introduces additional ethical and legal challenges related to privacy, consent, and the use of genetic data beyond its original purpose. As such, FGG represents an important extension of the reference sample framework in forensic identification, but its application requires careful regulation and integration within established forensic practices [89].

**Integration and Decision Framework**

In practice, forensic workflows adopt a hierarchical approach: attempt direct STR or mtDNA comparison to personal belongings; if unsuccessful, perform kinship analysis with first-degree relatives using autosomal STRs (and mtDNA/Y-STRs as needed); and if inconclusive, expand to more distant relatives with additional markers or SNP panels. Concordant results from multiple reference sources (e.g., matching both a sibling’s STR profile and a database hit) reinforce identification, whereas discordances prompt the re-evaluation of mutation probabilities or the possibility of sample contamination.

## 5. Types of Bone Samples for Genetic Identification

Sampling focuses on obtaining sufficient quantities of DNA to allow for further analysis [68]. Bone cells that contain DNA represent only 2% of the bone tissue, and these samples are often exposed to environmental factors such as temperature, humidity, and contaminants, which promote their degradation and, consequently, the fragmentation of DNA molecules [38,44,52,90,91,92]. Sampling should, thus, prioritize minimal destruction while maximizing the DNA yield [75,76,90,92,93,94].

Several studies describe a significant variability in DNA preservation among bones from different anatomical regions, which is essentially based on the porosity/presence of lacunae, (in)organic composition and the size of each bone [19,31,75,90,93,95,96,97].

Smaller and more porous bones tend to decompose more quickly than larger and denser bones [19,96]. Compact/cortical bones are good choices for DNA extraction due to their high bone density, which allows for better DNA preservation [95,98,99,100,101]. Teeth are also a good source of DNA, since dental hard tissues (enamel, dentine and cementum) are more resistant to degradation and sources of contamination [101]. While dental hard tissue contains less DNA, the pulp has traditionally been the best source of DNA, due to the higher number of cells, which are protected by enamel in the crown and cementum in the root. Additionally, 97% of enamel composition is inorganic material, making it more resistant to environmental influences over time, but less resistant to degradation. Dentine has more nuclear than mtDNA, in contrast to cementum, which has more mtDNA. However, overall, the nuclear DNA in cementum is still much higher than in dentine. Nuclear DNA recovered from dentine decreases over time due to pulp degradation, but, when recovered from cementum, nuclear DNA remains unaffected by age or dental disease, due to cementum’s highly mineralized cellular structure, which protects against environmental, microbial, and enzymatic damage. Nevertheless, the cementum layer is thin, so it is susceptible to contamination [99,100,101].

Several studies have compared the results obtained from different bone tissues (petrous, teeth, femur, tibia, calcaneus, talus, etc.) with different PMIs (post-mortem intervals), with the petrous bone showing the highest quantity and quality of DNA retrieved [31,75,90,97,101,102,103]. To enhance sampling options, recent studies have explored the feasibility of using smaller and more porous bones, such as phalanges, metacarpals, and metatarsals [19,98,104]. Results showed that small and trabecular bones yield a significant amount of DNA; therefore, they are also a great option for genetic analysis [19,104]. A comparison was also made between trabecular and compact bone; using two specific femur structures (epiphyses and diaphysis), it was shown that trabecular bone, due to its lower density, facilitates the extraction process through sample grinding, and trabecular bone provides more DNA than compact [98].

Considering this, the most commonly used bone structures for forensic genetic analysis are dense bones (petrous), long and compact/cortical bones (femur and tibia), and teeth [19,75,90,97,98,101]. However, it is essential to access each case individually, taking into account the context in which the samples were recovered, namely the burial (individual or mass grave) and environmental/geological conditions of this site, which might have a greater impact on DNA preservation than the (in)organic features of skeletal remains themselves [31,92,97]. Each case is unique, making it important to continuously understand the influence of environmental factors in skeletal remains and DNA preservation while searching for alternative skeletal elements that are suitable for each analysis [31,97]. Table 1 schematically describes the suitability of different skeletal human samples for DNA recovery.

## 6. Pre-Treatment Methods for Bone Samples

Since skeletal samples are often exposed to various sources of contamination and degradation, it is important to apply pre-treatment methods to obtain DNA of a sufficient quantity and quality for genetic analysis [31,92,93,101,105,106]. Thus, these samples require additional care compared to other matrices used in forensic contexts [92,105].

Ensuring minimal destruction of the sample is crucial to honor the respective individual, family and ancestors. In addition, skeletal samples are usually provided through museums, which also require a transparent methodology and procedures for sharing respective results and findings. For ethical, cultural and legal aspects, DNA recovery from skeletal samples is limited, making it essential to preserve as much of the anatomical structure as possible without damaging the existing DNA [94,101].

To remove contaminants from the bone surface, three decontamination methods can be applied: physical, chemical, and UV radiation [31,42,47,106,107]. Physical decontamination involves cleaning the bone surface using drills or sandpaper to remove external contaminants [19,31,42,105,108]. Grinding or cutting equipment operates at a low speed; however, this technique results in the destruction of part of the bone’s anatomical structure. An adaptation of this technique is micro drilling, which involves making small holes in specific areas of the bone or tooth to access the DNA without compromising the overall structural integrity [90,93,97,106,107,108]. Chemical decontamination uses solutions such as water, sodium hypochlorite, and/or ethanol to eliminate biological and chemical contaminants [19,105,108]. UV radiation decontamination involves exposing bone elements to ultraviolet light to eliminate surface contaminants [31,42,46,47,97,106]. These techniques allow for the removal of external contaminants without significantly altering the anatomical structure of the sample. They can be used individually or in combination [42,107]. However, they must be applied with caution, as excessive use can damage the DNA [31,90,106,109,110].

To facilitate the extraction process, the bone tissue is often pulverized to increase its contact surface with DNA extraction reagents [92]. To avoid sample overheating—and consequently, DNA degradation—liquid nitrogen is used [31,90,97,106]. Demineralization is a commonly adopted method after bone/tooth pulverization and prior to DNA extraction [101,105,107]. This technique involves the removal of the bone/tooth’s inorganic component, releasing the cells that contain DNA. Typically, multiple cycles of washing and overnight incubation with a chelating agent, such as ethylenediaminetetraacetic acid (EDTA), are used [105,107]. Discarding the supernatant helps to reduce contaminants in the sample. However, discarding the wash solution may also result in DNA loss. One solution to this problem was the incorporation of EDTA into the lysis buffers, reducing the need for washing steps [46,105]. Nonetheless, in more complex samples, pre-extraction demineralization may be used to improve both the quality and quantity of the obtained DNA [101,105,107].

Pre-treatment methods are essential to reduce or even eliminate the contaminants present in the samples, but they must be selected and adapted to the specific sample being analyzed [42,92,98]. These methods should be applied with moderation, as the goal is to eliminate contaminants—yet excessive use can lead to the destruction of the DNA present in the sample [92,93,110].

## 7. Methods for Extracting Nucleic Acids from Bone Samples

**Introduction**

The main purpose of DNA extraction is to obtain enough DNA copies but also minimize the extraction of associated inhibitors—proteins, lipids and other contaminants that can affect results by producing false negatives and unreliable outcomes [36,111,112]. The first experiment conducted with DNA extraction was accidentally developed in 1869 by Friedrich Miescher, a Swiss physician [113,114]. Throughout all these years, several samples and protocols have been tested, including specialization for skeletal samples [115,116,117].

The overall output from previous studies is that we either obtain more endogenous DNA quantity and precise results, or we save time and money: DNA extraction methods face barriers that hinder standardization [36,118]. Thus, the first and most important decision is defining the most appropriate method, considering the samples that will be analyzed and the main goal to achieve, so that protocols may be adapted if needed [111,119].

It is essential to ensure that cell and protein lysis occurs, releasing genetic material and enabling DNA purification [111,120,121]. Methods have evolved to use smaller quantities of a sample while still obtaining sufficient material for genomic analysis [36]. Euskirchen et al. (2021) showed that reducing the starting sample quantity and lysis duration can save valuable material and time without compromising DNA quality [120]. Forensic research has adopted some protocols that are commonly used in ancient genomic analysis due to their optimization for low-quantity and highly fragmented DNA, conditions that are frequently presented by skeletal human remains [15,122,123,124].

DNA purification removes contaminants, inhibitors and nucleases, preventing further degradation. Purification methods include silica-based, magnetic bead-based, salting-out and alcohol precipitation and microfluidic and high-throughput systems. A relevant method is silica binding, where DNA is purified by binding to silica in the presence of chaotropic salts, such as guanidinium thiocyanate: this promotes the separation of DNA from PCR inhibitors [112,125].

**Evolution of methods**

The study of DNA extraction has come a long way. The traditional phenol/chloroform/isoamyl alcohol (Ph-Chl-IA) method remains in use for its high DNA yield, despite being labor-intensive and hazardous [32,111,125]. Common extraction methods include silica-based methods, either in suspension or in columns [126]. One of the earliest methods was the silica-in-suspension technique by Boom et al. (1990), using guanidinium thiocyanate to bind DNA to silica particles [127]. The Chelex^®^ 100 method, one of the better time and resource consumption protocols being used in forensic contexts for quick and low-cost DNA extraction [128], focused on boiling and resin binding. Although it is a quick and low-cost protocol with minimal steps compared to silica or phenol–chloroform methods, it is not optimal for degraded samples, like human remains, since it provides a poor inhibitor removal; however, with adapted protocols (namely, an extended period of decalcification and an increased amount of Proteinase K at an extended incubation period), Chelex extraction might be successful with degraded human skeletal remains [122,129,130].

In March 2007, Rohland and Hofreiter optimized a silica column method for ancient DNA, combining EDTA demineralization and proteinase K digestion to effectively recover DNA from bones and teeth [117].

Considering the quality of the extracted material, different adaptations have been made in extraction protocols. One of the major steps is demineralization, which facilitates DNA liberation from mineralized areas of skeletal remains. The work developed by Loreille and colleagues in June 2007 offered a cost-effective approach for forensic and historical samples. It is focused on total demineralization (with higher concentrations of EDTA and Proteinase K, allowing for the complete dissolution of bone/tooth powder), which increases the amount of DNA extracted, as well as the size of the fragments. This requires minimal amounts of bone powder and faces the losses verified in the recovery of the supernatant after demineralization with reduced concentrations of EDTA and Proteinase K [131]. The protocol of Rohland and Hofreiter from July 2007 is scalable, quick, and requires minimal equipment; it is suitable for various sample sizes and types. It has been successfully applied to samples from caves, open sites, and permafrost environments, yielding amplifiable DNA for downstream applications like PCR [132,133]. Later, in 2013, Dabney and colleagues refined these techniques for ultra-short DNA fragments, which are critical in paleogenomics. The Dabney method (designed for ancient DNA) is effective for extracting short DNA fragments from degraded samples [134]. This modified approach could be promising for further forensic research [110], since it used silica in columns and a binding buffer (in a higher volume) containing guanidine hydrochloride, sodium acetate, and isopropanol. In 2019, Dabney and Meyer used silica columns (with an additional option of MinElut PCR Purification Kit), which were effective for extracting fragments of ~35 bp. In this work, there was a combination of EDTA with Proteinase K in lysis buffer and combination of GuHCl (guanidine hydrochloride) and isopropanol in binding buffer [130]. These studies were especially important because they addressed the central limiting factor in genetic identification from skeletal remains: the recovery of authentic, highly degraded DNA of a sufficient quantity and quality for downstream analysis.

In 2021, Xavier and colleagues compared the Loreille and Dabney methods, concluding that the Dabney method is more suitable for damaged or ancient samples, whereas the Loreille method is more suitable for recent and better-preserved samples [109].

To schematically describe the suitability of different and mostly studied DNA extraction protocols for degraded human skeletal remains, Table 2 focuses on the main protocols optimized for these types of samples, with the main range of fragments having a length that can be retrieved, while Table 3 focuses on the differences between silica-based extraction methods (DNA binding to silica through magnetic beads in suspension versus DNA binding to a silica membrane on a column by centrifugation). DNA binding to magnetic silica beads in suspension is currently the most widely used method of damaged DNA extraction [15,110,135].

More recently, commercial kits for purification and isolation were developed and allowed for automatization. They are quicker and require less manipulation, which reduces the risk of contamination, but they do not remove all the inhibitors. PrepFiler™ Express BTA (by Thermo Fisher, Massachusetts), NucleoSpin^®^ DNA Forensic (by Macherey-Nagel, Düren, Germany), QIAamp DNA Investigator Kit (by Qiagen, Hilden, Germany) and DNA IQ System (by Promega, Madison, USA) are commercial kits that have become widely used for degraded forensic samples, validated in comparative studies. Additionally, custom magnetic bead protocols and lab-on-chip microfluidic methods are being developed to improve efficiency and field applicability [111,117,129,136,137].

Nowadays, when it comes to recovering bone powder from ancient remains, it is common to use silica-based methods, but some procedures on protocols are not as useful as previously thought, even minimizing the amount of DNA obtained [138]. Reagents like Dithiothreitol (DTT) and N-phenacyl thiazolium bromide (PTB) are examples of this. DTT might introduce cuts in DNA structure and does not simplify protein lysis in the expected way, as Proteinase K is already doing this; PTB cannot disintegrate some molecules [138,139,140]. Furthermore, choosing reagents and procedures that allow for protein lysis and DNA liberation, without breaking even more DNA strands and enhancing the recuperation of short fragments, is crucial to improve the endogenous DNA yield and reliability in genetic authenticity, as it avoids biased conclusions or partial genetic profiles. This highlights that the choice of extraction method should depend on the condition of the sample and the specific genotyping application needed [110,111].

Moreover, the use of combined DNA extraction methods (automated and organic-based) might be highly advantageous, since it improves the overall information obtained and eases the construction of a consensus profile [32].

**Progress and Future Prospects**

Contamination persists as the main barrier to obtaining a high endogenous DNA yield that is free of inhibitors and exogenous DNA [136]. It is mandatory to compare different protocols and refine the existing techniques, namely enhancing recovery rates and developing more semi-automated workflows to improve efficiency and consistency in processing large numbers of samples and strategies to reduce the costs and time associated with DNA analysis, making it more accessible for various applications [36]. The development of more studies using different samples according to the origin and type of human remains is crucial to widen sampling possibilities in different contexts [119].

## 8. Genetic Marker Panels Used in Human Identification

**Genetic Markers**

Commonly used genetic markers include autosomal STRs [137] and lineage-based markers such as Y-STRs, Y-SNPs, and mitochondrial DNA [11,27,30,33,35,45]. However, these markers require reference samples or family pedigrees for comparison. In cases like mass disasters, missing people, or historical remains, reference data may be unavailable, for which ancestry-informative and phenotype-informative SNPs allow for human identification beyond conventional approaches [67,69,141,142,143].

STRs are commonly used in forensic science for individual identification and kin-ship analysis, due to their high polymorphism and discriminative power [50,137]. In contrast, SNPs are typically biallelic and individually less informative, but are the most prevalent form of genetic variation in humans, with low mutation rates, and enable higher multiplexing (simultaneous amplification and single-reaction analysis of multiple genetic markers) with shorter amplicons (often under 150 bp and potentially as short as 50–60 bp). Due to SNPs’ lower polymorphism, it takes a larger number of markers to achieve discrimination power that is comparable to STRs [123,143,144].

Insertion/deletion (InDel) polymorphisms are common genetic variations where one or more DNA bases are inserted or deleted from a sequence. InDels have a low mutation rate, a small amplicon size, are compatible with capillary electrophoresis (CE) and present fewer stutter artifacts (artifacts from DNA polymerase slippage that can obscure true alleles from minor contributors) [50]. Autosomal InDels are effective for identification and paternity testing, but may lack resolution in complex kinship cases. X-chromosome InDels, however, offer greater utility, due to their unique inheritance pattern, making them particularly informative for relationships like half-siblings or grandmother–granddaughter pairs [39,50,145,146].

Thus, STRs present high polymorphism, but SNPs and InDel makers have short amplicon sizes and present fewer stutter artifacts—artifacts from DNA polymerase slippage—that can obscure true alleles from minor contributors. This is relevant when dealing with degraded samples or samples with small amounts of DNA. Moreover, SNPs and InDels markers have a low mutation rate, making them applicable for kinship analysis and paternity tests and lowering the probability of false exclusion [50].

Interpreting DNA mixtures using STRs can also be challenging, especially in cases involving multiple individuals or imbalanced contributions. Key limitations include the preferential amplification of shorter alleles and the presence of stutter peaks. Additionally, degraded or low-quantity DNA samples are prone to stochastic effects during PCR, which can further complicate STR-based analysis [38,44,52].

Microhaplotypes (MHs) were introduced as forensic genetic markers in 2014. They are short DNA regions—typically 150 to 300 base pairs long—that contain two or more closely linked SNPs with varying allele frequencies. Unlike STRs, MHs are based on sequence polymorphisms and do not exhibit issues such as preferential amplification or stutter peaks, making them particularly useful for analyzing mixed DNA samples. However, because MHs are generally less polymorphic than traditional STR markers, a larger number of MH loci is needed to reach comparable levels of discrimination in forensic identification [143,147].

Mitochondrial DNA (mtDNA) is a key genetic marker in evolutionary and population studies, due to its high mutation rate, maternal inheritance, and lack of recombination. Its abundance in cells makes it especially useful when nuclear DNA exists in small quantities or is degraded, such as in aged or damaged samples, so that it cannot be tested or even detected. Moreover, mtDNA offers the advantage of accessing a broader range of reference samples from maternal relatives, as it allows for a comparison between the DNA recovered from the bone and the reference mtDNA profiles of potential maternal relatives to assess whether there is a match [40,58,148,149]. Although nuclear DNA generally offers more detailed results, an mtDNA profile is still valuable when no nuclear DNA can be recovered [26,27,33].

Table 4 schematically summarizes the suitability of genetic markers—InDels, SNPs, STRs, mtDNA, Y-chromosome and microhaplotypes—in degraded human skeletal samples in forensic contexts, considering their properties.

**Progress**

STRs are considered the gold standard for forensic kinship analysis. However, in cases with degraded samples, which is often the case for human skeletal remains, autosomal STR typing usually generates partial profiles that could not confirm kinship. Identity SNPs are, then, seen as a promising alternative. Furthermore, the combination of STRs and SNPs significantly improved the kinship analysis for degraded DNA samples.

The high sensitivity and ability to analyze short DNA fragments of massive parallel sequencing (MPS) technology is advantageous for challenging investigations [50,102,123,143]. Conventional STR PCR-CE typing (by capillary electrophoresis) has been replaced with PCR-MPS technology, namely in challenging bone samples. NGS enables high-throughput sequencing through MPS, with simultaneous processing of multiple samples using unique barcodes, providing high coverage, detailed sequence data, and high-level multiplexing. MPS has also improved forensic analysis by enabling the development of commercial kits for STR and mtDNA analysis; it has even expanded the use of SNPs—ancestry and phenotype markers—in human identification by overcoming previous limitations of single base extension methods [35,123,144,150,151]. Overall, PCR/MPS provide more complete genetic profiles than PCR/CE; combining both technologies is advantageous because one method can replace the markers lacking in the other, allowing for higher-quality typing [152].

**Genetic Marker Panels**

There are two main panels used worldwide: CODIS (Combined DNA Index System), combining 13 original STR loci from 1997 and later expanded to 20 loci in 2017, and the ESS (European Standard Set) panel, which initially included seven STR loci defined in 1999 [153], and was later expanded to 12 loci in 2009, when five additional loci were added [154,155,156].

The Codis panel was founded by the FBI (Federal Bureau of Investigation) and is routinely used in the USA [157]. Later, ESS was developed to harmonize and streamline cross-border forensic DNA profiling across Europe, particularly operating under the Prüm Treaty [153,158]. Combining commercial kits with Interpol’s recommendations [3], the European Network of Forensic Science Institutes (ENFSI) and the European DNA Profiling Group (EDNAP) introduced seven loci in 1999. These were selected based on their widespread use, with high discrimination power, and were compatible with the existing commercial kits. In 2009, five additional loci were added to enhance the power of discrimination, ensure compatibility with degraded samples, and fulfill the technical requirements of the Prüm Decision. This update aligns the ESS with the expanded CODIS core loci used in the United States and improves its international interoperability and database compatibility [153,159,160].

There are some STR multiplex PCR commercial kits designed for forensic human identification, such as PowerPlex (Promega) and GlobalFiler (Thermo Fisher Scientific). Thermo Fisher Scientific developed six pre-designed targeted sequencing panel kits that are ready-to-use products in NGS research—the “Ion AmpliSeq community panels”: Body Fluid Identification DNA and RNA, VISAGE-Basic Tool, DNA Phenotyping, PhenoTrivium, HID Y-SNP and MH-74 Plex [161]. Besides these applications, Thermo Fisher Scientific has developed five other panels for human identification (Precision ID Panels): mtDNA Whole Genome, mtDNA Control Region, GlobalFiler NGS STR, Identity (with SNPs), and Ancestry (with SNPs as well) [123,162].

**Conclusions**

To enable positive identification from skeletal remains in forensic, civil, or archeological contexts, authors must consider the sample type, preservation conditions and DNA quality when selecting an appropriate genetic marker panel. Such panels should comprise highly polymorphic, independent (unlinked and independently inherited loci) and well-characterized (for relevant populations to support accurate statistical calculations) markers with short amplicon sizes, ensuring high discriminatory power, robustness and reproducibility when working with limited or highly degraded DNA. Furthermore, these panels must allow for multiplexing, forensic validation and legal acceptance [11,163].

## 9. Analysis of Results and Statistical Evaluation

Interpreting DNA results from human skeletal remains requires a rigorous statistical framework that is capable of handling the specific challenges associated with degraded, low-template and often partial genetic profiles. These challenges include allelic drop-out, contamination risk, and limited quantity or quality of nuclear DNA. The objective is to translate such profiles into scientifically robust conclusions about identity, using models that quantify the strength of the evidence in a legally defensible way. Whether the comparison is direct (e.g., with a personal item or a known profile), indirect (through kinship with relatives) or lineage-based (e.g., mitochondrial or Y-chromosome DNA), all evaluations must rest on probabilistic principles [31,103,152].

The central statistical tool used in forensic identification is the Likelihood Ratio (LR) (Formula 1). The LR quantifies how much more (or less) likely the observed DNA profile is under one hypothesis versus another [164].

If the profiles are concordant at all tested loci and the profile is complete, the LR is often approximated by the inverse of the Random Match Probability (RMP), which is calculated using allele frequencies from an appropriate reference population. In such cases, RMPs as low as 1 in 10^8^ or 10^9^ are not uncommon when using a full set of autosomal STR markers, especially when the profile originates from a well-preserved bone sample.

The LR is calculated as the following formula [165]:

$$LR=\frac{P\left(DNA\;data/H_{1}\right)}{P\left(DNA\;data/H_{2}\right)}$$

In a direct comparison, the two hypotheses might be: H_1_, the bone sample originates from the reference individual, or H_2_, the bone sample originates from an unknown, unrelated person in the population.

In kinship-based comparisons, which are common in the identification of unknown human remains, the LR compares the probability of the observed genotypes under two competing familial scenarios. For example: H_1_, the bone profile belongs to the biological child of the reference individual, or H_2_, the bone profile belongs to an unrelated person.

The final LR is the product of locus-specific LRs, under the assumption of independence between loci. These are calculated based on population allele frequencies and expected genotype distributions for the tested relationship. For first-degree relatives (parent–child, full siblings), increasing LR values correspond to increasing support for the claimed relationship and are commonly interpreted within qualitative frameworks of evidential strength, in line with recommendations from the International Society for Forensic Genetics and the European Network of Forensic Science Institutes (ENFSI). For example, ENFSI guidelines provide indicative ranges in which LR values between 10^3^ and 10^4^ may be described as providing moderate to strong support, while values exceeding 10^4^ may be considered to provide strong support [166,167]. These categories, however, are intended for communication purposes and should be understood as part of a continuous scale, rather than as fixed thresholds. In forensic casework, LRs in kinship analyses are often supplemented with posterior probabilities, which are computed using Bayes’ theorem by incorporating a prior probability based on circumstantial or contextual information. In jurisdictions where posterior probabilities are used to support identification (such as in humanitarian or legal repatriation contexts), these values contribute to decision-making processes alongside other lines of evidence, as reflected in guidelines from Interpol [168]. However, both LR values and posterior probabilities should be interpreted within a case-specific framework, with the final assessment integrating all available genetic and non-genetic evidence.

For bone samples, especially when only degraded DNA is available, lineage markers such as mitochondrial DNA (mtDNA) or Y-STRs are often analyzed. These markers are useful when nuclear DNA is absent or insufficient for autosomal STR profiling. However, since such profiles are shared along maternal (mtDNA) or paternal (Y-STR) lineages, the evidentiary value of a match is necessarily lower than that of autosomal DNA. The strength of evidence is usually expressed as the inverse of the haplotype frequency in a relevant population database. Care must be taken when interpreting these results, especially when dealing with underrepresented populations or small databases, as frequency estimates may not be accurate or stable.

An additional challenge in kinship analyses involving bone remains is the possibility of mutations, particularly when STR loci show one or two mismatches in otherwise compatible profiles. STRs have known germline mutation rates, which are typically around 10^−3^ per generation, depending on the locus [169]. These are incorporated into statistical calculations so that the LR reflects the possibility of such discrepancies. For example, a single mismatch between a bone profile and a putative parent or child at a fast-mutating locus does not necessarily weaken the evidence against a biological relationship. Instead, mutation models, often locus-specific, are used to adjust the LR accordingly. However, multiple mismatches, especially at loci with low mutation rates, reduce the strength of the evidence for relatedness.

Given the degraded nature of DNA in many bone samples, stochastic effects such as allelic drop-out and drop-in are common. This makes standard (binary) interpretation models inadequate. Instead, probabilistic genotyping software (e.g., STRmix™, EuroForMix, DNA View, LRmix) has become essential. These programs employ statistical models to account for peak heights, stutter artifacts, drop-out, drop-in, degradation, and other PCR-related effects. They calculate the LR by integrating over all possible genotype combinations and assigning weights based on the observed data and assumed conditions (e.g., number of contributors, degradation levels). In particular, they allow the incorporation of continuous data, such as peak heights, providing more robust evaluations than traditional threshold-based methods. These tools are particularly valuable in skeletal DNA analysis, where samples often yield partial profiles or low-template results.

To ensure the validity and reproducibility of skeletal DNA results, rigorous quality assurance procedures are required. These include:

- Replicate extractions and PCRs from separate skeletal elements or aliquots to ensure profile concordance;
- Examination of peak height ratios, allelic balance, and stutter profiles;
- Confirmation of profile reproducibility across multiple amplifications;
- Careful contamination controls, including reagent blanks and negative controls.

In standard forensic practice, only DNA profiles confirmed by at least two independent extractions are reported as valid. Discordant profiles or those showing ambiguous peaks are either resolved through additional testing or excluded from interpretation.

In under-represented genetic population databases, limitations related to reduced statistical precision lead to biased random match probabilities and likelihood ratios. These databases may not consider population substructures, such as endogamy, which violates Hardy–Weinberg equilibrium assumptions, increasing the risk of false inclusions or exclusions. Allele frequencies may misrepresent how rare or common a genetic profile truly is, affecting random match probabilities and likelihood ratios by overstating or understating the evidential weight. The lack of representative data also decreases the accuracy of ancestry inference and kinship analysis. Forensic marker panels are less informative in under-represented populations, especially in complex mixtures or low-template DNA cases. This reduces the identification power and raises ethical and legal concerns, because the evidential weight may be uneven or more easily challenged in court, due to database inadequacy.

Finally, all DNA-based conclusions from bone analysis must be reported with scientific transparency and include the DNA profile(s) obtained, the statistical method used (LR, RMP, haplotype frequency), the reference populations and databases used, any corrections applied (e.g., mutation models) and any limitations of the analysis (e.g., drop-out, partial profile, contamination risk). The report should avoid speculative statements about activity level or the cause of DNA transfer. Instead, the geneticist must restrict their conclusions to the sub-source level (i.e., the origin of the DNA profile), leaving further inferences to the court or investigative authorities.

## 10. Cases of Historical and Scientific Relevance

Mass graves, armed conflicts and archeological excavations have not only demonstrated that genetic identification is possible, but they also reveal where methodologies succeed, where they fail, and what practitioners must anticipate. The following cases are examined not only for their outcomes but for the analytical decisions, operational challenges, and methodological lessons they produced.

There are several cases of disasters and wars in which corpses were buried in mass graves and the families of those individuals never got to mourn their family members. With the evolution of genetic techniques and the finding of those mass graves, there is now a possibility to identify those individuals and help to bring closure to their families.

Clandestine mass graves are frequently associated with mass executions, and they keep being found worldwide. In Iraq, more than 200 graves containing around 12,000 victims were found; in Bosnia, 94 mass graves and 337 superficial areas were retrieved, with around 17,000 human remains, of which almost half were identified; and in Mexico, 2.000 clandestine graves were found in 24 states with almost 3000 corpses, of which more than half were identified [170].

In Slovenia, the identification of victims buried in mass graves in the period after the Second World War represents a significant challenge, as it is estimated that 100,000 victims are distributed in approximately 600 mass graves. The first exhumations began in 1991, and the genetic identification started in 2006, with the successful identification of 32 of the 88 victims of the Konfin Shaft I Cemetery and three victims of the Storžič Cemetery [107]. The victims were found in different post-mortem conditions, which directly impacted the quality of the DNA extraction. Several human remains were analyzed, such as long bones (femurs and tibias), teeth, petrous bones, torso bones, meta-carpus, metatarsus and sesamoid bones. Priority was given to petrous bones, femurs, tibias, and teeth—consistent with the principle that compact and dense cortical bone provides better DNA preservation [31]. Metacarpals, metatarsals, and sesamoid bones were included when preferred elements were absent or too degraded, and smaller trabecular elements provided a supplementary yield, confirming findings that such bones yield meaningful DNA quantities despite a lower density. While this hierarchical selection strategy reduced analytical failure rates, the absence of ante-mortem references for many victims emerged as a critical limitation: kinship-based identification was only possible where living relatives could be traced, leaving a substantial fraction of remains unresolved despite the technical success of this methodology [107].

Bone density has been considered as a relevant factor in DNA preservation. The petrous bone, long bones and teeth are considered the most effective human remains to retrieve DNA in cases where much degradation occurred [31].

In 2015, a boat of migrants shipwrecked close to the Coast of Libya, taking hundreds of victims. The boat was retrieved in 2016, with 528 corpses and more than 30,000 human remains [171]. These remains were exposed to several environmental conditions, such as water submersion time and advanced decomposition. The retrieval of DNA from human remains submersed in salted water poses a difficult challenge for identification because the bone structure may be modified by these extreme conditions [171]. Different extraction methodologies were used together with several STR kits with different primers to maximize the DNA yield. The use of STR profile databases compiled from family members of reported missing people was the critical factor enabling identifications. Where it was possible to retrieve DNA, comparison against reference profiles provided positive identifications that would have been impossible by anthropological means alone, given the degree of disarticulation and decomposition [171,172]. However, the incompleteness of those reference databases meant that profiles successfully generated in the laboratory could not always be matched, leaving a portion of cases forensically unresolved.

In Vietnam, there is a large number of human remains belonging to armed conflicts in the twentieth century that were not yet identified. In this study [173], as nuclear DNA was scarce due to the degradation, techniques to retrieve mtDNA were performed. Hence, whole mitochondrial genome sequencing by NGS was chosen over control region sequencing by Sanger technology, since NGS tolerates shorter fragments and yields more phylogenetically informative data [173]. However, the profiles could not be converted into robust forensic identifications due to the absence of validated population-specific mtDNA reference databases for Southeast Asian populations, illustrating that analytical sophistication is insufficient when the supporting reference infrastructure is lacking.

In 1998, in Sweden, a Viking Age mass grave was found and an investigation using mtDNA analysis was conducted. It was possible to obtain 15 mtDNA profiles, and three of them were shared between the individuals. Most of the individuals were not maternally related, but it was possible to identify two pairs of mother–child or sibling relationships. It was also possible to assign a haplogroup to each sequence [174]. These findings illustrate both the strength and the limitation of mtDNA in archeological contexts: it can reconstruct maternal kinship networks and population affiliations, but cannot distinguish between mother–child and sibling pairs. The absence of nuclear or Y-chromosome markers represents a limitation that future studies on similar aged material should seek to address where preservation allows so.

Taken together, these cases demonstrate that no single protocol is universally optimal—taphonomic context dictates analytical strategy. Equally, the statistical infrastructure of forensic genetics (population databases, haplotype frequency tables, kinship software) emerges as a limiting factor that is as consequential as laboratory capability: in both the Vietnam and boat of migrants cases, technically successful results were forensically weakened by inadequate reference data, making the expansion of STR and mtDNA population coverage as urgent a priority as improving the extraction protocols. NGS/MPS proved superior in the most degraded scenarios, yet its value remains conditioned by validated bioinformatic pipelines and representative databases.

Between pioneering breakthroughs and routine methodologies, the genetic identification of human skeletal remains has grown in forensic contexts for either humanitarian or legal purposes. When anthropological or dental methods are insufficient, genetic kinship analysis often supplements traditional methods. These are some of the examples, among many, where genetic identification helped in disaster victim identification of human remains.

## 11. Ethical Considerations

Genetic human identification through skeletal remains involves not only methodological and analytical challenges but also significant ethical considerations that must be carefully addressed. Regardless of whether remains originate from forensic or archeological contexts, genetic analysis engages issues of human dignity, namely respect for the deceased and the obligation to minimize destructive sampling of often irreplaceable material. Consent represents a particularly complex aspect of this process, as it is frequently indirect or institutional, and obtained through judicial authorities, ethics committees, or governmental frameworks. In humanitarian and post-conflict scenarios, however, identification efforts often require active engagement with families and communities, where cultural, religious, and social perspectives must be considered, and where expectations regarding identification and repatriation may differ.

The storage, use, and sharing of genetic data also raise important ethical and legal concerns. Forensic DNA profiles must be handled under strict policies governing data protection, retention, and access, in order to prevent misuse and ensure confidentiality. Regulatory frameworks such as the General Data Protection Regulation (GDPR) establish key principles for data processing, including purpose limitation, proportionality, and security. These considerations are particularly relevant in the context of forensic DNA databases and transnational data exchange, where the harmonization of legal and ethical standards remains challenging.

Emerging approaches such as forensic genetic genealogy (FGG) further complicate the ethical landscape. By relying on genetic data from third-party individuals in genealogical databases, FGG raises questions regarding informed consent, privacy, and proportionality, particularly when applied to unidentified human remains. Additionally, ethical concerns arise when working with underrepresented or vulnerable populations, including indigenous communities, where issues of data ownership, cultural sensitivity, and historical misuse of genetic information must be carefully addressed. Therefore, the development of clear ethical guidelines and governance frameworks is essential to ensure that genetic identification practices are conducted responsibly, transparently, and with respect for both individuals and communities.

## 12. Conclusions and Future Perspectives

Genetic identification of human skeletal remains is challenged by low-yield, fragmented DNA and PCR inhibitors. Forensic research has improved its analytical techniques, combining STR and SNP typing, mtDNA analysis and MPS/NGS technologies. Only few reviews address aged skeletal remains analysis, but it is consensual that there is not a single method that performs optimally across all samples and preservation scenarios. Therefore, quantitative comparisons of performance metrics across techniques can help to establish methodological priorities, globally accepted guidelines and more efficient use of time and resources [172].

In this review, we highlight the main technological and methodological advances in the analysis of human skeletal remains with the purpose of genetic identification. The admissibility of DNA evidence in court has been one of the major fights [175], but there are still many limitations that must be overcome [163,176,177]: improved and suitable preservation techniques and bone type selection [14,132,178]; enhanced extraction protocols [133,179,180]; and a universally accepted framework that combines STR accuracy with SNP robustness and interpretative precision, supported by diverse population data and reliable models for complex and real-world samples [179,181,182,183,184]. These advances help to address ongoing challenges such as degraded samples and ethical considerations.

When performing genetic analysis on human remains, appropriate sampling strategies avoid material loss and maximize the DNA yield. Future research should prioritize minimally invasive and non-destructive sampling, including the integration of advanced imaging techniques such as computed tomography, to guide precise targeting of dense skeletal regions with high DNA preservation potential [13,135]. Coupling imaging-guided sampling with optimized extraction protocols and contamination-control measures would reduce the destructive impact, enhance the recovery of endogenous DNA, and support ethically responsible genetic identification across forensic, archeological, and historical contexts.

When considering mtDNA, Y-chromosome, STR and SNP markers, CE and MPS technologies, there is not a quantitative comparison of their performance metrics. Several studies have compared CE with MPS, namely through STRs; NGS with STR and SNP; and mtDNA with Y-chromosome, both with Sanger and MPS. mtDNA often succeeds in cases where autosomal STR typing fails due to DNA degradation and is more effective at discriminating low-DNA or highly degraded bones instead of Y-chromosome. Nevertheless, to allow high throughput and achieve high-discrimination power, MPS/NGS is far more advantageous than in CE on STR, SNP, Y-chromosome and mtDNA [69,173,183]. While STRs enhance individualization when DNA quality allows so, SNPs increase discrimination in sensitive and degraded samples. Recent and comparative studies thus mention autosomal STR typing as the usual first option, followed by mtDNA and SNPs, especially considering cold cases, historical investigations and mass disaster contexts.

Regarding the evolution of DNA extraction protocols from degraded samples, methods have evolved to extract smaller quantities while still obtaining sufficient material for genomic analysis [185]. Future research should consider automated and high-yield protocols: namely, hybrid workflows that combine damaged DNA techniques (e.g., specialized decalcification and purification steps for capturing ultrashort fragments and removing inhibitors that block downstream STR typing or sequencing) with forensic standards (for STR typing and human identification) [15].

Technology advancements, like NGS and targeted enrichment techniques, improve deeper information recovery, especially in degraded or poorly preserved samples. The combination of NGS-based typing with optimized sampling and extraction should increase the success rates from extremely degraded remains. Furthermore, interdisciplinary cooperation with complementary scientific and forensic disciplines, such as archeology and anthropology, enhances analytical strategies and improves the accuracy, reliability and contextual interpretation of results [10,11,12,31,82,138,186,187].

Beyond technological development, respect for the deceased must prevail. Each step foresees a meaningful outcome, particularly the provision of reliable identifications that can be communicated to families and affected communities. Despite these well-intended practices, we still need global and ethical guidelines that address the issues of storage, sampling, data management, responsibility, and oversight. Only through comprehensive consideration of these aspects and sustained collaboration among experts can genetic identification efforts be conducted efficiently, responsibly, and with full respect for human remains.

## Figures and Tables

**Table 1:** Organic and inorganic characteristics of human skeletal elements that influence DNA degradation, preservation, and recovery. Suitability for DNA recovery (high, medium, low) is based on a qualitative assessment of trends reported in the literature, considering factors such as DNA preservation, environmental protection, and reported recovery success.

Skeletal Samples		Organic Features	DNA Yield	Suitability for DNA Recovery
Petrous region of temporal bone		Dense and compact structure with reduced vascularization—minimize microbial entry and biochemical/environmental degradation	High nuclear and mtDNA yield—high number of osteocytes in a bone structure with low remodeling rate	High Limited use due to anatomical location and sampling constraints
Teeth	Enamel	More resistant to environmental influences and contamination, but less to degradation	Low	Low
	Dentine	More nuclear than mtDNA Nuclear DNA decreases over time due to pulp degradation	Low	Low
	Cementum	More nuclear and mtDNA than in dentine Nuclear DNA does not decrease over time Layer is thin, being susceptible to contamination	High	High
	Pulp	Higher number of cells, which are protected by enamel in the crown and cementum in the root	High	High
Femur, tibia and humerus		Compact/Cortical bones High density	High	High
Calcaneus Talus		Mix structure: spongy and compact	Low	Low
Phalanges Metacarpals Metatarsals Epiphyses		Trabecular and porous bones Low density	Medium	Medium Although it facilitates extraction process through sample grinding

**Table 2:** Suitability of the main extraction methods tested for degraded human skeletal samples, focused on differential DNA fragment lengths’ recovery.

Extraction Method/Protocol	Suitability for Degraded Skeletal Samples
Phenol/chloroform/isoamyl alcohol (Ph-Chl-IA)	Low—Inefficient for highly fragmented DNA and poor inhibitor control
Chelex^®^ 100	Low—Limited recovery of short fragments, boiling increases DNA fragmentation and lacks effective inhibitor removal
Loreille et al. (2007) [131]	Low to Medium—Complete demineralization with full dissolution of the sample. Low recovery of ultra-short DNA fragments (<143 bp) but able to increase total DNA yield
Rohland & Hofreiter (2007) [132]	Medium Only EDTA + proteinase K for bone digestion Binding DNA to silica via guanidinium thiocyanate for DNA purification BSA in PCR helps to overcome inhibitors
Dabney et al. (2013) [134]	High—Optimized for ultrashort aDNA fragments (<50 bp)
Rohland et al. (2018) [126]	High—Optimized for (ultra)short aDNA fragments (>25 bp)
Dabney & Meyer (2019) [130]	High—Optimized for highly degraded aDNA fragments (~35 bp)

**Table 3:** Suitability of silica-based extraction methods for degraded human skeletal samples.

Silica-Based Extraction	Advantages	Disadvantages	Suitability for Degraded Skeletal Samples
Silica in Suspension	Excellent recovery of short fragments (>25 pb) More efficient removal of inhibitors (reduced co-elution) More affordable	More procedures (including preparation of reagents) Time-consuming	High Method of choice in ancient DNA laboratories
Silica in Columns	Good recovery of short fragments (>35 bp) Efficient removal of inhibitors Practical Quick with few samples	High cost (commercial kits)	Moderate Mostly used in recent samples

**Table 4:** Suitability of different genetic markers for degraded human skeletal samples in forensic contexts.

Genetic Marker	Genetic Markers’ Suitability for Degraded Human Skeletal Samples in Forensic Contexts		
	Properties	Outcome	
InDels	Short amplicon sizes Low mutation rate Less informative	Kinship analysis Identification of degraded samples/with small amounts of DNA	Applicable with standard techniques X-chromosome InDels for complex kinship cases
SNPs			SNPs < 150 bp and potentially 50–60 bp Higher multiplexing with shorter amplicons Ancestry and phenotype informative
STRs	Longer fragments High polymorphisms High discriminative power	Limited Individual identification and kinship analysis	
mtDNA	High number of copies High mutation rate Maternal lineage Lack of recombination	High Identification in forensic and genealogical contexts	
Y	Paternal lineage	Limited	
MHs	150–300 bp with two or more closely linked SNPs Less polymorphic than traditional STR markers	Less discriminatory Suitable for mixed DNA samples	

## Data Availability

Data is contained within the article.

## Abbreviations

The following abbreviations are used in this manuscript:

NGS	Next-Generation Sequencing
MPS	Massive Parallel Sequencing
CE	Capillary Electrophoresis
STR	Short Tandem Repeats
SNP	Single Nucleotide Polymorphisms
Y-STR	Y-Chromosome STR
Y-SNP	Y-Chromosome SNP
mtDNA	Mitochondrial DNA
InDels	Insertion–Deletion Polymorphisms
MHs	Microhaplotypes
LRs	Likelihood Ratios
aDNA	Ancient DNA
CODIS	Combined DNA Index System
ESS	European Standard Set
FBI	Federal Bureau of Investigation
ENFSI	European Network of Forensic Science Institutes
EDNAP	European DNA Profiling Group
GDPR	General Data Protection Regulation
FGG	Forensic Genetic Genealogy
RMP	Random Match Probability
PCR	Polymerase Chain Reaction
EDTA	Ethylenediaminetetraacetic acid
LCN	Low Copy Number
PMI	Post-Mortem Interval
